# Development of clinical decision rules to predict recurrent shock in dengue

**DOI:** 10.1186/cc13135

**Published:** 2013-12-02

**Authors:** Nguyen Tien Huy, Nguyen Thanh Hong Thao, Tran Thi Ngoc Ha, Nguyen Thi Phuong Lan, Phan Thi Thanh Nga, Tran Thi Thuy, Ha Manh Tuan, Cao Thi Phi Nga, Vo Van Tuong, Tran Van Dat, Vu Thi Que Huong, Juntra Karbwang, Kenji Hirayama

**Affiliations:** 1 Department of Immunogenetics, Institute of Tropical Medicine (NEKKEN), Nagasaki University, 1-12-4 Sakamoto, Nagasaki 852-8523, Japan; 2 Department of Clinical Product Development, Institute of Tropical Medicine (NEKKEN), Nagasaki University, 1-12-4 Sakamoto, Nagasaki 852-8523, Japan; 3 Children’s Hospital, No. 2, 14 Ly Tu Trong Street, Ho Chi Minh City 70000, Vietnam; 4 Laboratory of Arbovirus, Pasteur Institute in Ho Chi Minh City, 167 Pasteur Street, Ho Chi Minh City 70000, Vietnam; 5 Department of Immunology & Microbiology, Pasteur Institute in Ho Chi Minh City, 167 Pasteur Street, Ho Chi Minh City 70000, Vietnam; 6 University of Medicine and Pharmacy at Ho Chi Minh City, 217 Hong Bang, District 5, Ho Chi Minh City 70000, Vietnam; 7 Center for Preventive Medicine, 24 Hung Vuong Street, Vinh Long 91000, Vietnam

## Abstract

**Introduction:**

Mortality from dengue infection is mostly due to shock. Among dengue patients with shock, approximately 30% have recurrent shock that requires a treatment change. Here, we report development of a clinical rule for use during a patient’s first shock episode to predict a recurrent shock episode.

**Methods:**

The study was conducted in Center for Preventive Medicine in Vinh Long province and the Children’s Hospital No. 2 in Ho Chi Minh City, Vietnam. We included 444 dengue patients with shock, 126 of whom had recurrent shock (28%). Univariate and multivariate analyses and a preprocessing method were used to evaluate and select 14 clinical and laboratory signs recorded at shock onset. Five variables (admission day, purpura/ecchymosis, ascites/pleural effusion, blood platelet count and pulse pressure) were finally trained and validated by a 10-fold validation strategy with 10 times of repetition, using a logistic regression model.

**Results:**

The results showed that shorter admission day (fewer days prior to admission), purpura/ecchymosis, ascites/pleural effusion, low platelet count and narrow pulse pressure were independently associated with recurrent shock. Our logistic prediction model was capable of predicting recurrent shock when compared to the null method (*P* < 0.05) and was not outperformed by other prediction models. Our final scoring rule provided relatively good accuracy (AUC, 0.73; sensitivity and specificity, 68%). Score points derived from the logistic prediction model revealed identical accuracy with AUCs at 0.73. Using a cutoff value greater than −154.5, our simple scoring rule showed a sensitivity of 68.3% and a specificity of 68.2%.

**Conclusions:**

Our simple clinical rule is not to replace clinical judgment, but to help clinicians predict recurrent shock during a patient’s first dengue shock episode.

## Introduction

Dengue virus infection has become an important global problem. It is quickly spreading to South America and Africa [1]. It is estimated that 20,000 patients die from this disease each year from among the 2.5 billion people who are at risk for dengue in over 100 countries [2]. The pathogenesis of dengue infection is not completely understood. Several mechanisms have been proposed, including a virulence factor [3-5], secondary infection [5], host genetic factors [6,7], host immune response [8-10], memory T-cell-mediated pathogenesis [8], suppressed Th1 and/or predominant Th2 responses [9], cytokine tsunami [10], anti-nonstructural 1 protein antibodies that cross-react with vascular endothelium [11] and host physiological factors [12].

Dengue disease, as defined according to the 1997 World Health Organization (WHO) classifications, ranges from asymptomatic or dengue fever to severe dengue hemorrhagic fever and dengue shock syndrome (DSS) [13]. Recently, in the 2009 revised dengue classification system proposed by Dengue Control, the WHO has added a classification of the severity of the disease according to the presence of dengue warning signs [14].

To date, there is no approved vaccine or antiviral drug to treat for this disease. Vector control, early appropriate treatment and educational programs are currently the only ways to reduce mortality and the global disease burden [14-17]. Mortality can reach 10% to 20% without early appropriate treatment [18]. Death due to dengue infection is reportedly 50 times higher in dengue patients with shock than in those without shock [19]. Among shock patients, approximately 30% have recurrent shock that reportedly affects the therapy protocol [20]. To the best of our knowledge, however, no previous report has described a tool for prediction of patients who will develop a second dengue shock episode. In this study, we developed a model to predict, at the time of the first shock episode, patients who would develop recurrent shock.

## Materials and methods

### Study design

The current study was performed at the Center for Preventive Medicine in Vinh Long province and the Children’s Hospital No. 2 in Ho Chi Minh City, Vietnam. The study design was a prospective cohort analysis of the clinical signs and laboratory test values of children (ages 6 months to 15 years) that were recorded around the time of the first shock episode. The entry criteria were suspected clinical dengue infection with proven dengue virus on the basis of laboratory evidence and hypovolemic shock that occurred between January 2002 and December 2007. Proven dengue virus infection was diagnosed if the virus was isolated, if RNA was detected by real-time PCR assay or if the serological assay was positive, as fully described in our previous publications [7,21,22]. DSS was classified according to the WHO 1997 classification criteria [13] without fulfilling the criterion for presence of thrombocytopenia [23]. Exclusion criteria were the presence of chronic illness or massive bleeding that required blood transfusion.

Around the time of shock, the relevant patient history regarding clinical symptoms and signs, as well as the laboratory parameters listed in Table 1, were recorded. These variables are widely used in our hospitals for the diagnosis and monitoring of dengue shock patients. Pleural effusion and ascites were detected by chest X-ray and ultrasound. A patient was defined as having developed recurrent shock if he or she had received adequate fluid according to the WHO 1997 guidelines for volume replacement [13] and had tachycardia, abnormal coolness of limbs and a pulse pressure ≤25 mmHg that had previously reached a level ≥30 mmHg [20].

**Table 1:** **Clinical characteristics, laboratory parameters and univariate analysis**^**a**^

**Characteristics**	**Single shock**	**Recurrent shock**	**Missing data (%)**	***P*-value**
	**(*N*** **= 318)**	**(*N*** **= 126)**		
Age (years)	10 (0.25 to 15)^b^	10 (0.5 to 15)	0	0.95
Females/total	171/318	73/126	0	0.43
Admission day	3.97 ± 1.20^c^	3.71 ± 1.08	1 (0.2)	0.035
Days of shock	4.95 ± 1.34	4.67 ± 1.38	14 (3.2)	0.054
Petechia	144/318 (45%)	61/126 (48%)	0	0.55
Tourniquet test	12/318 (4%)	5/126 (4%)	2 (0.5)	0.93
Nose/gum bleeding	40/318 (13%)	21/126 (17%)	0	0.26
Purpura/ecchymosis	98/318 (31%)	62/126 (49%)	0	<0.001
GI bleeding	36/318 (11%)	23/126 (18%)	0	0.052
Ascites/pleural effusion	114/318 (36%)	92/126 (73%)	1 (0.2)	<0.001
HCT (%)	43.01 ± 5.83	43.50 ± 5.50	3 (0.7)	0.43
PLTs (×10^3^/μl)	110.59 ± 56.10	96.63 ± 60.34	16 (3.6)	0.024
WBC count (×10^3^/μl)	5.55 ± 3.75	4.98 ± 2.73	44 (9.9)	0.141
Pulse pressure (mmHg)	18.41 ± 6.26	16.31 ± 7.24	0	0.001

^a^Days of shock, number of illness days prior to the first shock; GI, gastrointestinal; HCT, hematocrit; PLTs, platelets; WBC, white blood cell. Admission day, fever days prior to admission and day 1 of illness were assigned as the day of fever onset. ^b^Median (minimum, maximum) calculated using the Mann–Whitney *U* test for continuous variables not normally distributed. ^c^Mean ± SD calculated using Student’s *t*-test for continuous variables normally distributed.

The minimal required sample size was determined by rule of thumb, in which at least ten patients per group were required for each included predictive variable [24,25]. We aimed to use less than ten variables to build prediction models for ease of use in clinical practice. The required sample size was at least 100 participants per group. This study was approved by the institutional ethical review committees of the Institute of Tropical Medicine, Nagasaki University, and the Pasteur Institute in Ho Chi Minh City. Written informed consent from all participants’ parents or guardians was required upon enrollment.

### Univariate and multivariate analyses

The primary outcome variable was recurrent shock diagnosis at the time of hospital discharge. Univariate analysis was performed using SPSS software version 16.0 (SPSS, Inc, Chicago, IL, USA). Measures of skewness and kurtosis were used to test the normal distribution of continuous variables. Student’s *t*-test was used for continuous variables normally distributed, and the Mann–Whitney *U* test was used for continuous variables that were not normally distributed. χ^2^ analysis was used for categorical variables. A difference with a *P* value <0.05 was considered significant. Multivariate logistic regression analysis was used to find the independent predictors of recurrent shock using MedCalc version 11.0 statistical software (MedCalc Software, Ostend, Belgium).

### Missing data analysis

Missing data ranged from 0% to 9.9%. Little’s missing completely at random test [26] was performed using SPSS software version 16.0, which showed a nonsignificant result (*P* > 0.10), suggesting that the data were missing completely at random. Therefore, multiple imputation algorithms were used to analyze the missing data using NORM software version 2.03 [27]. The multiple logistic regression model was employed to input missing data.

### Preprocessing variable selection

To increase the performance of the prediction model [28], a preprocessing method was employed to reduce the number of variables using Weka 3.7.7 software [29]. We used the assessment method in WrapperSubsetEval [30] combined with the best first search method in the forward direction [31] and the learning scheme of our logistic regression prediction model (Logistic). A ten-fold cross-validation method was used with the number of folds set at five and the seed set at one.

### Training and validation of the prediction model

**Figure 1:**
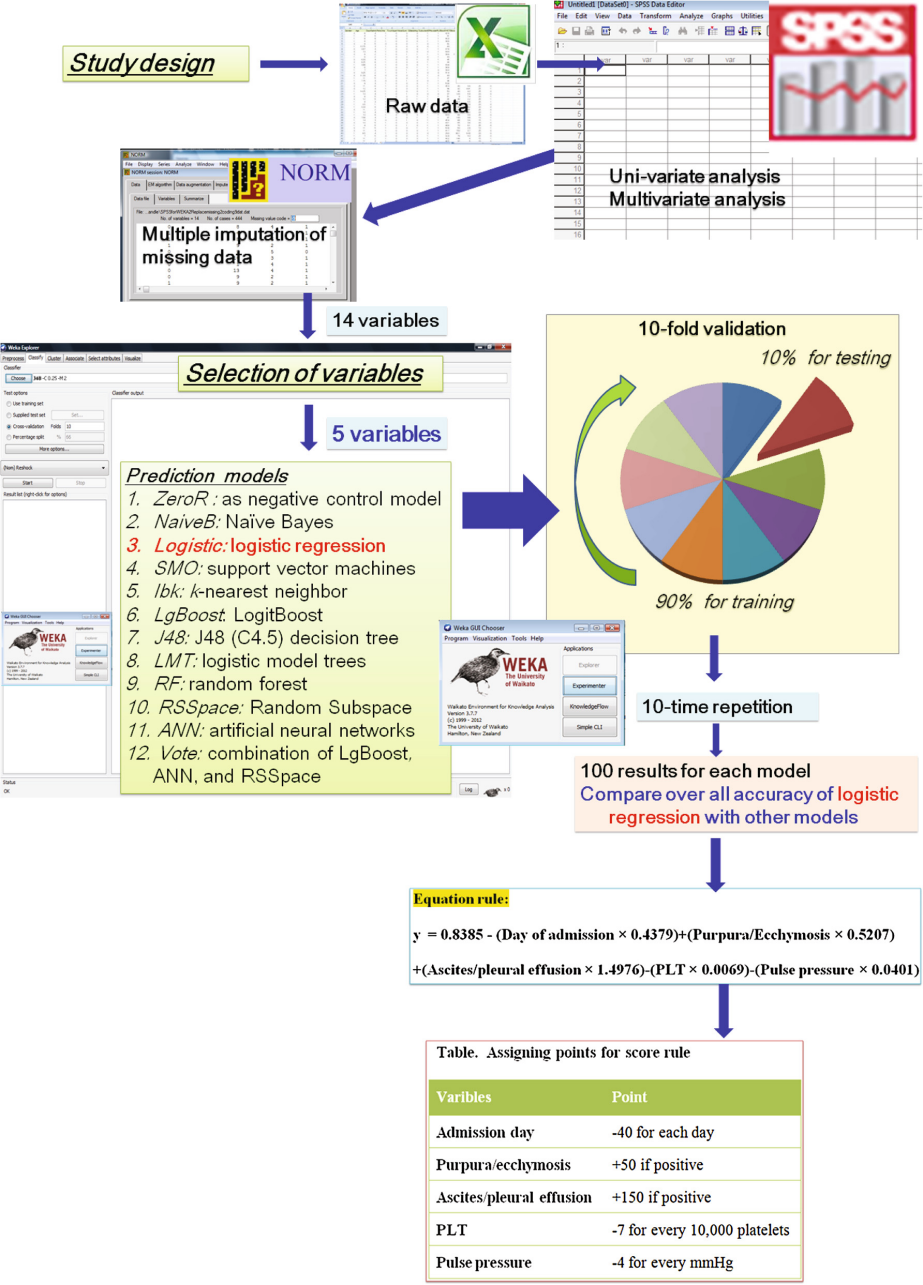
**Flowchart of the development of models for prediction of recurrent shock during a patient’s first dengue shock episode.** PLT, platelet.

Because a simpler rule would be preferred by clinicians, particularly in remote areas, we built a simple traditional rule using a logistic regression model [32]. Logistic was built and compared to ZeroR, a baseline model without predictive power, to identify the predictive power of the Logistic model. The overall performance of the data-mining model was assessed by calculation of the area under the curve (AUC) from the receiver operating characteristic (ROC) curve [33]. Weka 3.7.7 software [29] was used to train and validate all models in a ten-fold validation strategy with ten rounds of ten repetitions as previously described [34,35]. Briefly, the whole data set was randomly split into ten equal subsets (Figure 1). For ten times, nine subsets were used to train the model and the remaining subset was used to validate the AUC of each model. The cross-validation was repeated 10 times to yield a total of 100 AUC values for each model. The overall accuracy of these models, represented by the mean and standard deviation of 100 AUC values, was compared using the corrected, resampled *t*-test [36]. Differences between models with *P* values <0.05 were considered significant.

Initially, we used the default settings of the Weka software to build the prediction model. Next, we optimized parameters to calculate the highest AUC value. The full description of parameter-setting is presented in Additional file 1.

Score points were derived by multiplying and rounding regression coefficients to calculate the lowest integer, then simplifying them to achieve a scoring system. The optimal cutoff value was chosen as the q value of the ROC curve, at which sensitivity equals specificity.

### Comparison of logistic regression prediction model with the other ten prediction models

Ten binary prediction models (artificial neural network (ANN), *k*-nearest neighbors algorithm (*k*NN), C4.5 decision tree (J48), LogitBoost classification algorithm (LgBoost), logistic regression tree (LRT), naïve Bayes classifier (NaiveB), random forests (RF), random subspace (RSSpace), sequential minimal optimization (SMO) for support vector machines and a combination of ANN, LgBoost and RSSpace (Vote)) were built as described above. The overall accuracy of these rules was compared using the mean and standard deviation of 100 AUC values per model by using the corrected, resampled *t*-test [36].

## Results

### Patient characteristics and univariate analysis

A total of 444 proven dengue patients with shock, including 318 patients (72%) with a single shock episode and 126 (28%) with recurrent shock, were enrolled (Table 1). No deaths were recorded in the study. There were no significant differences in age and gender between the control (single shock) and recurrent shock DSS groups. The mean admission day (mean number of illness days prior to admission, and day 1 of illness was assigned as the day of fever onset) for the recurrent shock group was significantly lower than that of the single shock group (*P* < 0.05). The mean of the day of shock (illness days prior to the first shock) was slightly lower in the recurrent shock group than in the single shock group (*P* = 0.054). For the clinical and laboratory parameters, purpura/ecchymosis (*P* < 0.001), ascites/pleural effusion (*P* < 0.001) and lower platelet count (*P* < 0.05) and narrow pulse pressure (*P* < 0.001) were found more commonly in the recurrent shock group than in the single shock group. Gastrointestinal bleeding was closely associated with recurrent shock (*P* = 0.052). No significant differences between the two groups were found for petechia, tourniquet test, nose/gum bleeding, hematocrit and white blood cell count.

### Multivariate analysis and development of clinical decision rule

Because the clinical rule should be reliable and easy to apply [32], we aimed to develop a simple rule to predict the recurrent shock patients using the logistic regression model. After the preprocessing method of variables reduction, nine variables, including age, admission day, day of shock, petechia, purpura/ecchymosis, gastrointestinal bleeding, ascites/pleural effusion, platelet count, and pulse pressure, remained and were analyzed using a multivariate logistic regression model. The results in Table 2 show that admission day, purpura/ecchymosis, ascites/pleural effusion, blood platelet count, and pulse pressure, independently correlated with recurrent shock in a multivariate model (*P* < 0.05).

**Table 2:** **Multivariate logistic regression model used to predict dengue shock syndrome and nonshock cases**^**a**^

**Predictors**	**OR (95% CI)**	**Adjusted OR (95% CI)**	***P*-value**
Age	1.01 (0.95 to 1.08)	1.05 (0.98 to 1.13)	0.1756
Admission day^b,c^	0.82 (0.69 to 0.99)	0.66 (0.52 to 0.83)	0.0041
Day of shock	0.91 (0.77 to 1.07)	0.91 (0.76 to 1.08)	0.3476
Petechia	1.13 (0.75 to 1.71)	0.78 (0.46 to 1.30)	0.3381
Purpura/ecchymosis^c^	2.17 (1.43 to 3.32)	1.78 (1.11 to 2.86)	0.017
GI bleeding	1.75 (0.99 to 3.09)	1.37 (0.73 to 2.58)	0.1290
Ascites/pleural effusion^c^	0.23 (0.14 to 0.42)	0.24 (0.13 to 0.43)	0.0001
PLT (×10^3^/μl)^c,d^	0.99 (0.99 to 0.99)	0.99 (0.99 to 0.99)	0.0148
Pulse pressure (mmHg)^c,d^	0.96 (0.93 to 0.98)	0.96 (0.93 to 0.99)	0.0163

^a^CI, confidence interval; GI, gastrointestinal; OR, odds ratio; PLT, platelet. *Dengue shock syndrome* refers to dengue hemorrhagic fever, and *nonshock cases* refers to dengue fever.

^b^OR represents the decremental odds of recurrent shock for every unit decrease of one day in admission days. ^c^Variables were selected for developing the clinical rules. ^d^OR represents the incremental odds of recurrent shock for every unit increase of 1,000 platelets per microliter or every 1 mmHg increase in pulse pressure.

**Figure 2:**
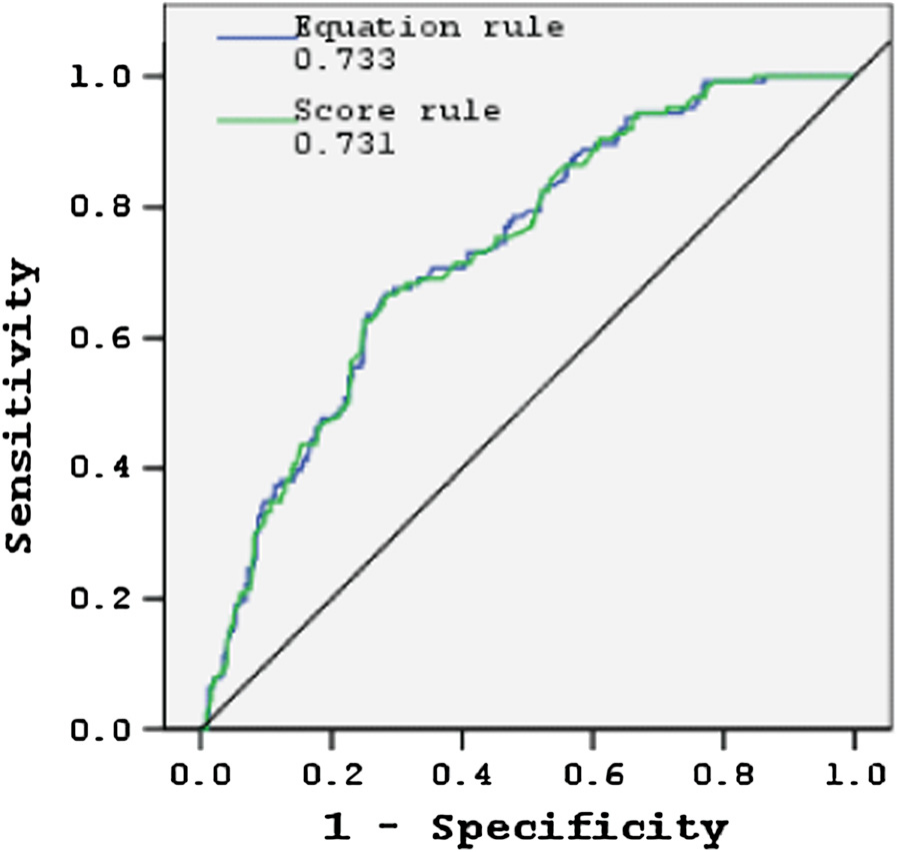
**Receiver operating characteristic curves of two traditional logistic prediction rules for prediction of recurrent shock during a patient’s first dengue shock episode.** The areas under the receiver operating characteristic curves were 0.733 for the equation rule and 0.731 for the scoring rule.

Five variables (admission day, purpura/ecchymosis, ascites/pleural effusion, blood platelet count and pulse pressure) were further trained and validated using a ten-fold validation strategy with ten rounds of repetition [34,35] using Weka 3.7.7 software [29]. Next, scores were derived by multiplying and rounding regression coefficients to calculate the lowest integer, then simplifying the coefficients to achieve a scoring system (Table 3). The total score for patients in the whole data set ranged from −406 points to +53 points. Figure 2 shows the performance of the logistic models built by using the equation and the scoring systems, revealing identical accuracy with AUCs at 0.73 when the whole data set was applied. Using a cutoff value greater than −154.5, the simple point rule revealed a sensitivity of 68.3% and a specificity of 68.2%.

**Table 3:** **Assigning score values for clinical decision rule**^**a**^

**Variables**	**Points**^**b**^
Admission day	−40 for each day
Purpura/ecchymosis	+50 if positive
Ascites/pleural effusion	+150 if positive
PLT	−7 for every 10,000 platelets
Pulse pressure	−4 for every 1 mmHg

^a^PLT, platelet. ^b^Derived from the following equation: *y* = 0.8385 − (day of admission × 0.4379) + (purpura/ecchymosis × 0.5207) + (ascites/pleural effusion × 1.4976) − (PLT × 0.0069) − (pulse pressure × 0.0401).

### Comparison of 11 prediction models

In this study, 11 popular data-mining methods (ANN, *k*NN, J48, LgBoost, LRT, Logistic, NaiveB, RF, RSSpace, SMO and Vote) were trained and compared to the baseline ZeroR model to identify the predictability power of the built models. Five variables (admission day, purpura/ecchymosis, ascites/pleural effusion, blood platelet count and pulse pressure) were computed using a ten-fold validation strategy. The results showed that all 11 prediction models possessed AUC values between 0.645 and 0.730 (Additional file 2). Compared to the baseline ZeroR method, the 11 prediction models all showed an ability to predict recurrent shock (*P* < 0.05). Among these models, the Vote model demonstrated the highest AUC at 0.730, followed by LgBoost (0.720), NaiveB (0.705), Logistic (0.703), LRT (0.703), *k*NN (0.696), SMO (0.695), ANN (0.695), RF (0.694), RSSpace (0.694) and J48 (0.645). Compared with the other four best models (Vote, LgBoost, NaiveB and Logistic), the J48 model was significantly outperformed in overall accuracy.

## Discussion

Medical doctors often initiate fluid resuscitation by using normal saline solution or Ringer’s lactate solution. Most children with DSS recover from shock with this treatment regimen; however, 28% to 30% have recurrent shock that requires administration of colloidal solutions and more intensive care [20] (Table 1). Therefore, a clinical decision rule is needed to predict recurrent shock during a patient’s first dengue shock episode. In this study, we defined a simple, practical, relatively accurate clinical decision rule (sensitivity and specificity of 68%) that can be applied at the bedside [32] to predict recurrent shock in children hospitalized for a first dengue shock episode. The rule contains a list of items with a detailed scoring system and does not require complicated mathematical computation.

Our univariate analysis revealed many factors associated with recurrent shock. There were more signs of purpura/ecchymosis, gastrointestinal bleeding, ascites/pleural effusion, low platelet count and narrow pulse pressure in recurrent shock patients compared with patients who had a single shock, similarly to the severity signs of dengue infection [2]. A short duration of fever prior to admission was associated with recurrent shock, suggesting rapid progression of the disease in recurrent shock patients. The mechanism of this phenomenon is unknown, thus further studies are required to clarify it.

Several other data-mining techniques, such as ANN, *k*NN, J48, LgBoost, LRT, NaiveB, RF, RSSpace, SMO and Vote, recently have been developed and used to help the clinician make decisions [37-42]. Though the traditional Logistic model has been used extensively to develop decision rules, other modern techniques are underutilized in clinical practices. It has been proposed that intensive computer-based data-mining classifiers outperform the traditional classification methods in several data sets [43-47]. However, this superiority has not been obvious in other data sets [48,49]. Thus, it is important to compare several models in order to find an optimal clinical decision-making model for a particular prediction [50]. In our present study, we simultaneously developed, tested and compared the traditional Logistic model with ten common prediction models for critical care in dengue infection. Our results show that all 11 models had significant power to predict, around the time of first dengue shock episode, which patients would develop a second shock episode. Five models (Vote, LgBoost, NaiveB, Logistic and LRT) had AUCs >0.7, which is considered an acceptable discrimination level [51]. Among these five models, no prediction rule had superior overall accuracy compared to the other rules. Furthermore, no prediction models were significantly better than the relatively simple logistic regression model in terms of overall accuracy (AUC).

There are several limitations of this study. First, we analyzed data from only two hospitals in southern Vietnam. Therefore, the results would have been different if we had used data from other hospitals, particularly those in other countries, where the clinical characteristics, epidemiology and outcomes of dengue infection are different. Second, the overall accuracy of the final model was not so high, thus more markers are needed to improve the efficacy of the rule in future. Third, we did not include treatment variables at the time of first dengue shock episode (such as initial fluids and length of stay in the ICU), Pediatric Risk of Mortality or Sequential Organ Failure Assessment score variables (such as heart rate, respiratory rate and creatinine level), which might have improved the accuracy of the prediction rule. In addition, we used only one software package to build all prediction models. Other software could produce different results regarding the best model. Thus, further prospective studies using data from different regions are required for external validation of the results of this study.

## Conclusions

Recurrent shock occurs in about 28% of dengue-infected patients with shock. We derived a simple clinical decision rule with a sensitivity and specificity of 68%, which could help clinicians treating dengue patients during the early stage of a first shock episode to predict the development of recurrent shock. The usefulness of this decision rule needs to be validated by several independent studies in the future.

## Key messages

•Several prediction models were capable of predicting reshock in children who had a first dengue shock (AUC >0.7).

•The simple traditional logistic regression model derived from five factors (admission day, purpura/ecchymosis, ascites/pleural effusion, blood platelet count and pulse pressure) provided relatively good accuracy with an AUC of 0.73.

## Abbreviations

ANN: Artificial neural network; AUC: Area under the curve; DF: Dengue fever; DHF: Dengue hemorrhagic fever; DSS: Dengue shock syndrome; J48: C4.5 decision tree; kNN: *k*-nearest neighbor; LgBoost: LogitBoost; LRT: Logistic regression tree; NaiveB: naïve Bayes classifier; RF: Random forests; RSSpace: Random subspace, ROC, receiver operating characteristic; SMO: Sequential minimal optimization.

## Competing interests

The authors have no competing interests with commercial or other affiliations.

## Authors’ contributions

NTH and KH conceived and planned the study. NTH, NTHT, TTNH, NTPL, PTTN, TTT, HMT, CTPN, VVT, TVD, VTQH, JK and KH carried out the study. NTH, NTHT, TTNH, NTPL, VTQH, JK and KH analyzed the data and wrote the paper. NTH, NTHT, TTNH, NTPL, TTT, HMT, CTPN, VVT, TVD, VTQH and KH contributed reagents and materials. All authors read and approved the final manuscript.

## Supplementary Material

Additional file 1 Parameters of the Weka software were optimized to obtain the highest area under the curve value for each model. Click here for file

Additional file 2 Head-to-head comparisons of area under the receiver operating characteristic curves of different models for predicting recurrent shock during a patient’s first dengue shock episode. Click here for file
