# Supplementary material for: Development of clinical decision rules to predict recurrent shock in dengue
Source: Crit Care. 2013 Dec 2;17(6):R280. doi: 10.1186/cc13135 (PMC4057383; doi:10.1186/cc13135)
Supplement: Additional file 1 — Parameters of the Weka software were optimized to obtain the highest area under the curve value for each model. [file cc13135-S1.doc]

**Additional file 1**

**Parameters setting to have the highest value of AUC for each model**

**1/ Artificial neural networks**

GUI -- Brings up a gui interface. This will allow the pausing and altering of the neural network during training. False

debug -- If set to true, classifier may output additional info to the console. True

decay -- This will cause the learning rate to decrease. This will divide the starting learning rate by the epoch number, to determine what the current learning rate should be. This may help to stop the network from diverging from the target output, as well as improve general performance. Note that the decaying learning rate will not be shown in the gui, only the original learning rate. If the learning rate is changed in the gui, this is treated as the starting learning rate. False

hiddenLayers -- This defines the hidden layers of the neural network. This is a list of positive whole numbers. 1 for each hidden layer. Comma seperated. To have no hidden layers put a single 0 here. This will only be used if autobuild is set. There are also wildcard values 'a' = (attribs + classes) / 2, 'i' = attribs, 'o' = classes , 't' = attribs + classes.: a

learningRate -- The amount the weights are updated. 0.3

momentum -- Momentum applied to the weights during updating. 0.251

nominalToBinaryFilter -- This will preprocess the instances with the filter. This could help improve performance if there are nominal attributes in the data. True

normalizeAttributes -- This will normalize the attributes. This could help improve performance of the network. This is not reliant on the class being numeric. This will also normalize nominal attributes as well (after they have been run through the nominal to binary filter if that is in use) so that the nominal values are between -1 and 1 True

normalizeNumericClass -- This will normalize the class if it's numeric. This could help improve performance of the network, It normalizes the class to be between -1 and 1. Note that this is only internally, the output will be scaled back to the original range. True

reset -- This will allow the network to reset with a lower learning rate. If the network diverges from the answer this will automatically reset the network with a lower learning rate and begin training again. This option is only available if the gui is not set. Note that if the network diverges but isn't allowed to reset it will fail the training process and return an error message. True

seed -- Seed used to initialise the random number generator.Random numbers are used for setting the initial weights of the connections betweem nodes, and also for shuffling the training data. 0

trainingTime -- The number of epochs to train through. If the validation set is non-zero then it can terminate the network early 87

validationSetSize -- The percentage size of the validation set.(The training will continue until it is observed that the error on the validation set has been consistently getting worse, or if the training time is reached). 0

If This is set to zero no validation set will be used and instead the network will train for the specified number of epochs.

validationThreshold -- Used to terminate validation testing. The value here dictates how many times in a row the validation set error can get worse before training is terminated. 20

GUI -- Brings up a gui interface. This will allow the pausing and altering of the nueral network during training.

* To add a node left click (this node will be automatically selected, ensure no other nodes were selected).

* To select a node left click on it either while no other node is selected or while holding down the control key (this toggles that node as being selected and not selected.

* To connect a node, first have the start node(s) selected, then click either the end node or on an empty space (this will create a new node that is connected with the selected nodes). The selection status of nodes will stay the same after the connection. (Note these are directed connections, also a connection between two nodes will not be established more than once and certain connections that are deemed to be invalid will not be made).

* To remove a connection select one of the connected node(s) in the connection and then right click the other node (it does not matter whether the node is the start or end the connection will be removed).

* To remove a node right click it while no other nodes (including it) are selected. (This will also remove all connections to it)

.* To deselect a node either left click it while holding down control, or right click on empty space.

* The raw inputs are provided from the labels on the left.

* The red nodes are hidden layers.

* The orange nodes are the output nodes.

* The labels on the right show the class the output node represents. Note that with a numeric class the output node will automatically be made into an unthresholded linear unit.

Alterations to the neural network can only be done while the network is not running, This also applies to the learning rate and other fields on the control panel.

* You can accept the network as being finished at any time.

* The network is automatically paused at the beginning.

* There is a running indication of what epoch the network is up to and what the (rough) error for that epoch was (or for the validation if that is being used). Note that this error value is based on a network that changes as the value is computed. (also depending on whether the class is normalized will effect the error reported for numeric classes.

* Once the network is done it will pause again and either wait to be accepted or trained more.

Note that if the gui is not set the network will not require any interaction.

**K-nearest neighbours**

KNN -- The number of neighbours to use. 111

crossValidate -- Whether hold-one-out cross-validation will be used to select the best k value. False

debug -- If set to true, classifier may output additional info to the console. True

distanceWeighting -- Gets the distance weighting method used. No distance weighting

meanSquared -- Whether the mean squared error is used rather than mean absolute error when doing cross-validation for regression problems. False

nearestNeighbourSearchAlgorithm -- The nearest neighbour search algorithm to use (Default: weka.core.neighboursearch.LinearNNSearch). LinearNNSearch

windowSize -- Gets the maximum number of instances allowed in the training pool. The addition of new instances above this value will result in old instances being removed. A value of 0 signifies no limit to the number of training instances. 0

**J48**

binarySplits -- Whether to use binary splits on nominal attributes when building the trees: True

collapseTree -- Whether parts are removed that do not reduce training error: True

confidenceFactor -- The confidence factor used for pruning (smaller values incur more pruning). 0.25

debug -- If set to true, classifier may output additional info to the console. False

minNumObj -- The minimum number of instances per leaf. 9

numFolds -- Determines the amount of data used for reduced-error pruning. One fold is used for pruning, the rest for growing the tree. 3

reducedErrorPruning -- Whether reduced-error pruning is used instead of C.4.5 pruning: False

saveInstanceData -- Whether to save the training data for visualization: True

seed -- The seed used for randomizing the data when reduced-error pruning is used. 1

subtreeRaising -- Whether to consider the subtree raising operation when pruning. False

unpruned -- Whether pruning is performed. True

useLaplace -- Whether counts at leaves are smoothed based on Laplace. True

useMDLcorrection -- Whether MDL correction is used when finding splits on numeric attributes. True

**LMT**

convertNominal -- Convert all nominal attributes to binary ones before building the tree. This means that all splits in the final tree will be binary.

debug -- If set to true, classifier may output additional info to the console. True

errorOnProbabilities -- Minimize error on probabilities instead of misclassification error when cross-validating the number of LogitBoost iterations. When set, the number of LogitBoost iterations is chosen that minimizes the root mean squared error instead of the misclassification error. True

fastRegression -- Use heuristic that avoids cross-validating the number of Logit-Boost iterations at every node. When fitting the logistic regression functions at a node, LMT has to determine the number of LogitBoost iterations to run. Originally, this number was cross-validated at every node in the tree. To save time, this heuristic cross-validates the number only once and then uses that number at every node in the tree. Usually this does not decrease accuracy but improves runtime considerably.True

minNumInstances -- Set the minimum number of instances at which a node is considered for splitting. The default value is 15.

numBoostingIterations -- Set a fixed number of iterations for LogitBoost. If >= 0, this sets a fixed number of LogitBoost iterations that is used everywhere in the tree. If < 0, the number is cross-validated. The default value is -1.

splitOnResiduals -- Set splitting criterion based on the residuals of LogitBoost. There are two possible splitting criteria for LMT: the default is to use the C4.5 splitting criterion that uses information gain on the class variable. The other splitting criterion tries to improve the purity in the residuals produces when fitting the logistic regression functions. The choice of the splitting criterion does not usually affect classification accuracy much, but can produce different trees. The default value is False.

useAIC -- The AIC is used to determine when to stop LogitBoost iterations. The default is not to use AIC.False.

weightTrimBeta -- Set the beta value used for weight trimming in LogitBoost. Only instances carrying (1 - beta)% of the weight from previous iteration are used in the next iteration. Set to 0 for no weight trimming. The default value is 0.

**LogitBoost**

classifier -- The base classifier to be used. decision stump

debug -- If set to true, classifier may output additional info to the console. True

likelihoodThreshold -- Threshold on improvement in likelihood. -1.7976

numFolds -- Number of folds for internal cross-validation (default 0 means no cross-validation is performed). 0

numIterations -- The number of iterations to be performed. 10

numRuns -- Number of runs for internal cross-validation. 1

seed -- The random number seed to be used. 1

shrinkage -- Shrinkage parameter (use small value like 0.1 to reduce overfitting). 1.0

useResampling -- Whether resampling is used instead of reweighting. False

weightThreshold -- Weight threshold for weight pruning (reduce to 90 for speeding up learning process). 100

**Logistic regression**

Default setting,

Debug: False

Ridge value in the log-likelihood 0.0001

Maximum number of iterations to perform:4

useConjugateGradientDescent -- Use conjugate gradient descent rather than BFGS updates; faster for problems with many parameters. True

**LogitBoost**

binarySplits -- Whether to use binary splits on nominal attributes when building the trees: True

collapseTree -- Whether parts are removed that do not reduce training error: True

confidenceFactor -- The confidence factor used for pruning (smaller values incur more pruning). 0.25

debug -- If set to true, classifier may output additional info to the console. False

minNumObj -- The minimum number of instances per leaf. 9

numFolds -- Determines the amount of data used for reduced-error pruning. One fold is used for pruning, the rest for growing the tree. 3

reducedErrorPruning -- Whether reduced-error pruning is used instead of C.4.5 pruning: False

saveInstanceData -- Whether to save the training data for visualization: True

seed -- The seed used for randomizing the data when reduced-error pruning is used. 1

subtreeRaising -- Whether to consider the subtree raising operation when pruning. False

unpruned -- Whether pruning is performed. True

useLaplace -- Whether counts at leaves are smoothed based on Laplace. True

useMDLcorrection -- Whether MDL correction is used when finding splits on numeric attributes. True

**Naïve Bayes:**

debug: false;

display model in old format: false;

use Kernel Estimator: true; use supervised discretization: false

**Random forest**

debug -- If set to true, classifier may output additional info to the console. True

maxDepth -- The maximum depth of the trees, 0 for unlimited. 2

numExecutionSlots -- The number of execution slots (threads) to use for constructing the ensemble. 1

numFeatures -- The number of attributes to be used in random selection (see RandomTree).0

numTrees -- The number of trees to be generated. 20

printTrees -- Print the individual trees in the output True

seed -- The random number seed to be used. 1

**Random SubSpace**

classifier -- The base classifier to be used. REPTREE

debug -- If set to true, classifier may output additional info to the console. True

numExecutionSlots -- The number of execution slots (threads) to use for constructing the ensemble. 1

numIterations -- The number of iterations to be performed. 100

seed -- The random number seed to be used. 1

subSpaceSize -- Size of each subSpace: if less than 1 as a percentage of the number of attributes, otherwise the absolute number of attributes. 0.45

**SMO**

buildLogisticModels -- Whether to fit logistic models to the outputs (for proper probability estimates). True

c -- The complexity parameter C. The default value is 1.

checksTurnedOff -- Turns time-consuming checks off - use with caution. False

debug -- If set to true, classifier may output additional info to the console. True

epsilon -- The epsilon for round-off error (shouldn't be changed). The default value is 1. 0E-12

filterType -- Determines how/if the data will be transformed. No normalization/standardization

kernel -- The kernel to use. PolyKernel

cacheSize 250007; checksTurnedOff: False; debug: false; exponent 1.0; useLowerOder: False

numFolds -- The number of folds for cross-validation used to generate training data for logistic models (-1 means use training data).

randomSeed -- Random number seed for the cross-validation. 2

toleranceParameter -- The tolerance parameter (shouldn't be changed). 0.001

**Voting:**

Multiple classifiers were implemented to get the best AUC value.

LogitBoost, Artificial neural networks, and Random Subspace were proven to get the best of vote model.

The parameters were identical to individual model as described above.
