# Supplementary material for: Development of clinical decision rules to predict recurrent shock in dengue
Source: Crit Care. 2013 Dec 2;17(6):R280. doi: 10.1186/cc13135 (PMC4057383; doi:10.1186/cc13135)
Supplement: Additional file 2 — Head-to-head comparisons of area under the receiver operating characteristic curves of different models for predicting recurrent shock during a patient’s first dengue shock episode. [file cc13135-S2.doc]

**Additional file 2**

***Comparison of prediction models.*** *Head to head comparisons of area under receiver operating characteristic curves (AUCs) of different models for predicting recurrent shock during a patient’s first dengue shock episode.*

| ***Models AUC(SD)*** | ***ZeroR*** | ***NaiveB*** | ***Logistic*** | ***SMO*** | ***IBk*** | ***LgBoost*** | ***J48*** | ***LMT*** | ***RF*** | ***RSSpace*** | ***ANN*** | ***Vote*** |
| --- | --- | --- | --- | --- | --- | --- | --- | --- | --- | --- | --- | --- |
| ***ZeroR***  ***0.500(0.000)*** | _ | <^a^ | < | < | < | < | < | < | < | < | < | < |
| ***NaiveB***  ***0.705(0.075)*** | >^b^ | _ | NS | NS | NS | NS | > | NS | NS | NS | NS | NS |
| ***Logistic***  ***0.703(0.075)*** | > | NS | _ | NS | NS | NS | > | NS | NS | NS | NS | NS |
| ***SMO***  ***0.695(0.079)*** | > | NS | NS | _ | NS | NS | NS | NS | NS | NS | NS | NS |
| ***IBk***  ***0.696(0.083)*** | > | NS | NS | NS | _ | NS | NS | NS | NS | NS | NS | NS |
| ***LgBoost***  ***0.720(0.076)*** | > | NS | NS | NS | NS | _ | > | NS | NS | NS | NS | NS |
| ***J48***  ***0.645(0.078)*** | > | < | < | NS | NS | < | _ | NS | NS | NS | NS | < |
| ***LMT***  ***0.703(0.073)*** | > | NS | NS | NS | NS | NS | NS | _ | NS | NS | NS | NS |
| ***RF***  ***0.694(0.071)*** | > | NS | NS | NS | NS | NS | NS | NS | _ | NS | NS | NS |
| ***RSSpace***  ***0.694(0.085)*** | > | NS | NS | NS | NS | NS | NS | NS | NS | _ | NS | NS |
| ***ANN***  ***0.695(0.073)*** | > | NS | NS | NS | NS | NS | NS | NS | NS | NS | _ | < |
| ***Vote***  ***0.730(0.072)*** | > | NS | NS | NS | NS | NS | NS | NS | NS | NS | > | _ |

*^a^*The AUC of the prediction model on the left was significantly lower than that of the one on the right.

*^b^*The AUC of the prediction model on the left was significantly higher than that of the one on the right.

NS, not significant
